# Impact of an extended light regimen imposed during nursery period on the performance and lipid metabolism of weanling pigs

**DOI:** 10.5713/ab.24.0270

**Published:** 2024-10-24

**Authors:** Guangfan Liu, Fen Su, Xingyue Zou, Xingming Yang, Liang Tian

**Affiliations:** 1 College of Animal Science and Technology, Nanjing Agricultural University, Nanjing, 210095, China; 2 College of Resources and Environmental Sciences, Nanjing Agricultural University, Nanjing, 210095, China

**Keywords:** Fat deposition, Melatonin, Photoperiod, Pigs, Weaning

## Abstract

**Objective:**

This study aimed to assess the impact of a prolonged photoperiod on the growth performance and lipid metabolism of weaned piglets.

**Methods:**

Twenty-four piglets weaned at 28 days of age were randomly dichotomized into two groups that were alternatively subjected to either long photoperiod (LP) group (16 L:8 D) or short photoperiod (SP) group (10 L:14 D) for 42days. Four replicates of three animals per replicates were used per experimental treatment.

**Results:**

Our results demonstrated that prolonged photoperiod increased piglet body weight, average daily weight gain (ADG), backfat thickness (BF), backfat index during the nursery period, and increased ADG, average daily feed intake (ADFI), and decreased the F/G of piglets during the experiment days 29 to 42. Meanwhile, we observed LP piglets’ plasma melatonin, growth hormone and serotonin levels were decreased at 14 d and 42 d compared to SP piglets. Moreover, up-regulated mRNA or protein expression of PPARγ and CEBPα, and lower mRNA or protein expression of MTR1, ATGL, HSL, PPARα, and CPT1α, were observed in back subcutaneous fat of LP group compared with that of SP group. Significant increases were observed in the mRNA or protein contents of lipogenic genes, including *C/EBPα*, *SREBP-1c*, *ACCα*, and *FAS*, in the liver of LP piglets, whereas *CPT1α* and *ACOX1* mRNA levels and PPARα and MTR1 protein expression were significantly downregulated in LP group compared to SP group. Extended photoperiod also increased lipid content in longissimus dorsi muscle that was associated with higher mRNA or protein levels of SREBP-1c, ACCα, FAS, Pref1, and LPL, decreased mRNA or protein contents of LeptinR, MTR1, HSL, and ACOX1.

**Conclusion:**

Together, these findings suggest that there is an advantage, in terms of growth performance and fat deposition, in imposing a prolonged light program (16-h light/d) on nursery piglets to alleviate the negative aspects of weaning stress.

## INTRODUCTION

Weaned piglets require a significant amount of energy for the growth and development during the nursery period [1]. However, the stress caused by weaning leads to a decrease in feed intake, and therefore lower energy intake [2]. As a result, a large amount of body fat is mobilized to meet the energy requirements, which is extremely detrimental to the health and growth potential of weaned piglets [3]. The low body fat content of piglets decreases the ability of piglets to withstand cold. Furthermore, the decreased fat deposition in weaned piglets has been demonstrated to be a significant factor limiting the growth performance of pigs in the subsequent growing-finishing period [4].

Recently, studies on the regulation of fat deposition or growth in weaning piglets mainly focused on manipulating dietary composition by supplementing dietary fat [5]. Nevertheless, the utilization of light program management has been widely adopted to enhance the growth performance of livestock. Several studies have reported that Jinjiang cattle subjected to a long photoperiod (LP) (16 h light and 8 h darkness) show significantly increased dry matter intake (DMI) and higher backfat thickness (BF) [6]. Moreover, it has been demonstrated that extended photoperiod (16 h of light per day) may contribute to greater live weight and carcass weight in growing-finishing pigs than those subjected to a short photoperiod (SP) (8 h of light/d) [7]. For weaned piglets, multiple studies have shown that an extended photoperiod (23 hours of light per day) leads to heightened feed intake, reduced energy requirements for maintenance, and enhances growth performance in the early post-weaning stage [8]. Thus, these findings suggest exposure to an extended light program may impact energy metabolism and production performance in domestic animals. However, the effects of prolonged photoperiod on lipid metabolism and growth performance of piglets at the whole nursery phase are still not well understood.

Therefore, this study was designed to characterize the effects of a prolonged photoperiod (16 h light/d vs 10 h light/d) on growth characteristics, blood lipid and hormone profiles, expression of lipid metabolism-related genes, and lipid accumulation in nursery pigs. This study provides clues for the use of light regimen management within the nursery phase to alleviate the pigs’ weaning stress.

## MATERIALS AND METHODS

### Ethics statement

All experimental procedures conducted in this study received approval from the Laboratory Animal Care and Use Committee of Nanjing Agricultural University with the project number SYXK2021-0043.

### Animals and experimental treatments

A total of 24 cross breed male piglets (Landrace×Large White×Duroc), weaned at an average age of 28 days with an average weight of 8.39±0.13 kg, were randomly dichotomized into two groups: the LP group (n = 12, 16 h light from 07:00 to 23:00 and 8 h darkness) and the SP group (n = 12, 10 h light from 07:00 to 17:00 and 14 h darkness). Two sets of piglets were accommodated in separate environmentally controlled rooms, each residing in concrete floor pens with a bedding layer consisting of approximately 4.0 cm of wood shavings, at the Research Facility of Nanjing Agricultural University. Four replicates (blocks) of three animals per replicates were used per experimental treatment. The average temperature of the rooms was set at 25°C±2°C during the nursery period. Lighting was provided using 4 light emitting diode (LED) tri-proof lights (LED-220-T8-32-01; OPPLE, Guangdong, China) per barn. The lights were suspended at a height of 2.3 m from the ground, and they were arranged evenly above the test pens of each compartment. To measure the light intensity (lux), a Mavolux 5032B USB lux meter (Gossen Photo and light measurement GmbH, Nuremberg, Germany) was used at 5 points in every pen (Front, middle, back, left, and right locations). Measurements were performed at approximately the height of pigs’ eyes (20 cm from the ground). Mean values of 110±3 lux in the long and 110±3 lux in the shot photoperiod compartments were determined. The entire experimental duration spanned a period of 42 days in total. During the 6-week nursery period, the pigs had free access to water and a standard grain-based weaner diet. The dietary formulation for the nursery phase responded to the nutritional requirements of growth development, based on data from the National Research Council (NRC) (2012). The composition and nutrient levels of the diet are shown in Table 1.

### Data collection and sampling

To determine weight gain animals were weighted in the beginning (day 1, after weaning), day 14, day 28, and day 42 of the experiment. Feed intake was determined by subtracting the leftovers at the end of each day from the initial feed quantity. For the back-fat index (a ratio of back-fat thickness to body weight) detection, animals were measured for back-fat thickness (BF) in the beginning (day 1, after weaning) was assessed using A-mode ultrasonography (Renco, Rockledge, FL, USA) as previously described [9], and measured with vernier calipers after slaughter at day 42 of the experiment. The piglets’ blood samples were collected in 5-mL sterile heparinized vacuum tubes (Greiner, Frickenhausen, Germany) from the vena cava at day 14, and day 42 of the experiment, between 07:00 and 09:00. The plasma was promptly isolated via centrifugation at 3,500×g for 15 minutes at 4°C, then preserved at −20°C for subsequent analysis. The tissue samples from back subcutaneous fat (BSF), liver, and longissimus dorsi muscle (LDM), obtained immediately following slaughter at day 42, were rinsed thoroughly in cold phosphate-buffered saline (PBS) solution and then cut into approximately 6-cm^2^ pieces. Tissues were flash-frozen in liquid nitrogen and stored at −80°C until further processing.

### Plasma lipid and hormone assay

Plasma levels of glucose, triglycerides (TG), non-esterified fatty acids (NEFA), total cholesterol (CHOL), high-density lipoproteins (HDL), and low-density lipoprotein (LDL) were individually measured by using Beckman AU680 (Beckman-Coulter, Brea, CA, USA), following the methods previously described [10]. For blood hormone assessment, a commercially available porcine enzyme-linked immunosorbent assay (ELISA) kit (Shanghai Enzyme-Linked Biotechnology, Shanghai, China) was utilized to measure plasma growth hormone (GH), leptin, insulin, melatonin, and serotonin content, respectively, according to the manufacturer’s instructions as previously reported [6]. Each plasma sample underwent duplicate analysis within a singular assay. For plasma insulin analysis, results with an intra-assay coefficient of variation (CV) of 3.0% to 6.0% were acceptable. For GH, leptin, melatonin, and serotonin analysis, results were considered to be acceptable with a CV of less than 5%.

### Lipid profiles assay

Tissue samples from BSF, liver, and LDM were mechanically homogenized in ice-cold PBS using a T18 laboratory digital ULTRA-TURRAX Package disperser (IKA, Shanghai, China), respectively. Following dilution with an equal volume of PBS, homogenized samples were utilized for the analysis of TG and NEFA. The concentrations of TG and NEFA were determined using a Porcine TG or NEFA ELISA kit, respectively (RD SYSTEMS, Minneapolis, MN, USA), following the manufacturer’s instructions as previously reported [11]. The protein content of each sample was assessed using the Pierce BCA Protein Assay Kit (Thermo Scientific, Waltham, MA, USA), subsequently employed to normalize the concentrations of TG or NEFA.

### Histopathology and oil red O staining

Tissue samples (n = 8 from each group) from the BSF liver, and LDM were collected immediately after slaughter, and then fixed in 4% formaldehyde and subsequently embedded in paraffin, respectively. The paraffin blocks were cut into tissue sections (5 μm) using RM2016 Biological Tissue Slicer (Leica Instruments, Shanghai, China). Then, hematoxylin and eosin (H&E) staining of tissue sections were evaluated by light microscopy (Nikon ECLIPSE E100; Nikon, Tokyo, Japan). For oil red O staining assessment, frozen tissues from 8 piglets in each group were embedded in tissue-Tek O.C.T compound (SAKURA, Torrance, CA, USA) on dry ice, and frozen sections were cut at a thickness of 7 μm with a cryostat (Thermo Fisher HM525; Thermo Fisher, Waltham, MA, USA) and mounted on glass slides. After staining with oil red O solution (Sigma-Aldrich, St. Louis, MO, USA), slides were subsequently counterstained with hematoxylin and coverslipped using an aqueous mounting media (Sigma-Aldrich, USA). Digitized images were acquired using a NanoZoomer Slide Scanner (Hamamatsu, Shizuoka, Japan). Image analysis was performed using the Image-Pro Plus6.0 software from Media Cybernetics (Rockville, MD, USA).

### Real-time quantitative polymerase chain reaction analysis

Total RNA was extracted from tissue samples (n = 12 from each group) by Trizol (Invitrogen, Carlsbad, CA, USA), and quantified with Spectrophotometer NANODROP-2000 (Thermo, Waltham, MA, USA). cDNA (2 μg) was synthesized with the reverse transcription kit (TaKaRa, Tokyo, Japan). Real-time quantitative polymerase chain reaction (PCR) was conducted on the QuantStudio 7 Flex system (ABI, Waltham, MA, USA) with the following program: step 1, 95°C for 30 s; step 2, 95°C for 5 s, 60°C for 30 s; step 3, 95°C for 15 s, 60°C for 1 min; and 95°C for 15 s, with 40 cycles of Step 2. Amplification was performed in a 25 μL reaction system containing specific primers (Table 2) and SYBR Premix Ex Taq II (TaKaRa, Japan). Primers were synthesized by Sangon Biotech (Shanghai, China). Relative gene expression was calculated using the comparative Ct method with the formula 2^−ΔΔCt^ [12]. The levels of mRNA were normalized in relevance to glyceraldehyde-3-phosphatedehydrogenase (*GAPDH*).

### Western blotting analyses

Total protein from frozen tissues was extracted using radio immunoprecipitation assay (RIPA) lysis buffer (KeyGen Biotechnology, Nanjing, China) containing a protease and phosphatase inhibitor cocktail (Sigma, Saint Louis, MO, USA) by procedures as previously described [11]. The protein concentrations were quantified by a BCA assay kit (KeyGen Biotechnology, China). Protein samples (50 μg) were separated by sodium dodecyl sulfate-polyacrylamide gel electrophoresis and then transferred onto PVDF membranes (Merck Millipore, Darmstadt, Germany). After blocking with 5% fat-free milk for 1 h at room temperature, membranes were incubated with primary antibodies including Rabbit anti-C/EBPα (D56F10, 1:1,000 dilution; Cell Signaling Technology, Boston, MA, USA), PPARα (sc-900, 1:1,000 dilution; Santa Cruz Biotechnology, Texas, TX, USA), PPARγ (ab209350, 1:1,000 dilution; Abcam Biotechnology, Cambridge, UK), MTR1 (A13030, 1:1,000 dilution; ABclonal Technology, Wuhan, China), SREBP-1c (sc-366, 1:1,000 dilution; Santa Cruz Biotechnology, USA), and GAPDH (10494-1-AP, 1:1,000 dilution; Proteintech Group, Chicago, IL, USA) antibody overnight at 4°C, followed by incubation with Goat anti-rabbit immunoglobulin G (IgG) horseradish peroxidase (HRP)-conjugated secondary antibody (HAF008, dilution 1:2,000; RD SYSTEMS, USA) for 1 h at room temperature. Proteins were visualized with ECL chemiluminescence reagents (KeyGen Biotechnology, China), and then the blots were quantified using Amersham Image Quant 800 (Cytiva, Wilmington, DE, USA). Band density was normalized according to the GAPDH content.

### Quantification of lipase activity

Tissues from the BSF, liver, and LDM were homogenized in ice-cold cell and tissue lysis buffer (Beyotime Biotechnology, Nanjing, China) using T18 digital ULTRA-TURRAX Package disperser (IKA, China), respectively. The homogenate was centrifuged at 13,000 g for 5 min at 4°C. The supernatants were recovered and stored in aliquots at −80°C until further use. Adipose triglyceride lipase (ATGL) and hormone-sensitive lipase (HSL) activities were determined in the supernatant via enzyme-linked immunosorbent assay kits (Enzyme-linked Biotechnology Co. Ltd, Shanghai, China) according to the manufacturer’s instructions as previously described [13].

### Statistical analysis

All the data were analyzed using SPSS Statistics 26.0 software (IBM SPSS, Armonk, NY, USA). Each weaned piglet was considered an experimental unit. Statistical differences between the LP group and the SP group were analyzed using Independent-Samples T Test. Results were expressed as mean with standard error of the mean. A p-value <0.05 was considered statistically significant.

## RESULTS

### Piglet performance

The effects of prolonged photoperiod on the growth performance of weaned piglets were shown in Table 3. In the 1 to 42 days period, the body weight, average daily gain (ADG), BF, and backfat index of pigs in the LP group were significantly greater than that in the SP group (p<0.05), and the average daily feed intake (ADFI) and the F/G (ADFI/ADG) between LP and SP groups were not significantly different. But in the 29 to 42 days period, the ADG and ADFI in the LP group were significantly improved (p<0.05), and F/G in the LP group were significantly decreased (p<0.05).

### Influence of prolonged photoperiod on plasma hormone and lipid profiles

By studying the effects of prolonged photoperiod on plasma lipids and plasma hormones (Tables 4 and 5), our results showed that compared with the SP group, the levels of TG (p = 0.096) and NEFA (p = 0.067) of piglets in the LP group had an increasing trend at 42 d, but there was no significant difference between the two groups at 14 d. Moreover, the levels of serotonin, melatonin and GH in LP group were significantly decreased at 14 d and 42 d (p<0.05), but there were no significant differences in insulin, and leptin between the two groups.

### Effect of prolonged photoperiod on lipid content in metabolically active tissues

**Figure 1:**
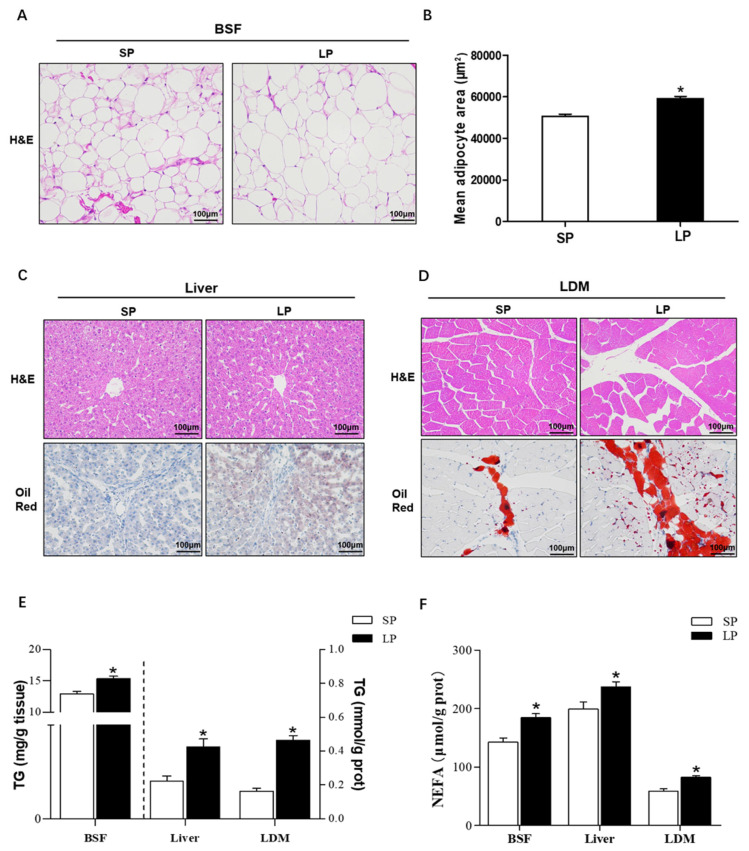
Effect of prolonged photoperiod on lipid content in metabolically active tissues. (A) Representative image of H&E staining in the subcutaneous fat and mean adipocyte area size (n = 8). (B) Representative image of H&E and oil red O staining in the liver and LDM (n = 8). (C) The level of triglycerides (n = 12). (D) The level of free fatty acids (n = 12). Results are expressed as mean±standard error of the mean. H&E, hematoxylin and eosin; LP, long photoperiod group; SP, short photoperiod group; BSF, back subcutaneous fat; LDM, longissimus dorsi muscle. * p<0.05; ** p<0.01.

To study the influence of prolonged photoperiod on tissue fat deposition, we determined the lipid content of the BSF, liver, and LDM (Figure 1). The HE staining analysis of BSF tissues showed (Figure 1A, 1B) that the adipocyte sizes of weaned piglets in the LP group were bigger than that in the SP group (p<0.05). Moreover, oil red O staining shows that prolonged photoperiod promotes lipid droplet enrichment in the liver and LDM tissue (Figure 1C, 1D). In the quantitative analysis of TG and NEFA in the tissues (Figure 1E, 1F), piglets in the LP group had significantly higher TG and NEFA levels in BSF, liver, and LDM tissues than that in the SP group (p<0.05).

### Effects of prolonged photoperiod on the expression of lipid metabolism-related genes, the activity of lipolytic lipase in subcutaneous fat

**Figure 2:**
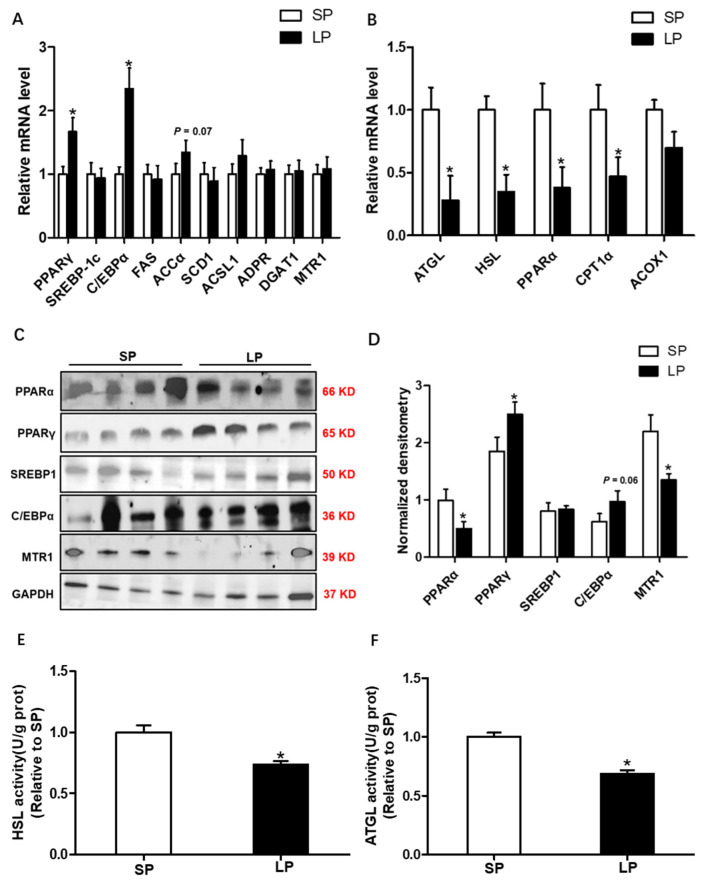
Effects of prolonged photoperiod on the expression of lipid metabolism-related genes, the activity of lipolytic lipase in subcutaneous fat. (A) The relative mRNA level of lipid synthesis genes *PPARγ, SREBP-1c, C/EBPα, FAS, ACCα, SCD1, ACSL1, ADPR, DGAT1*, and *MTR1* in LP and SP groups. (B) The relative mRNA level of lipolysis genes *ATGL, HSL, PPARα, CPT1α*, and *ACOX1* in LP and SP groups. (C) Western blot analyses were performed to detect the changes in *PPARα, PPARγ, SREBP-1c, C/EBPα*, and *MTR1*. (D) Densitometric analysis of corresponding proteins in (C) by normalization to GAPDH as an internal control. (E) Hormone-sensitive lipase activity. (F) Adipose triglyceride lipase activity. All values are presented as means±standard error of the mean (n = 12). Representative Western blot is presented. LP, long photoperiod group; SP, short photoperiod group. * p<0.05; ** p<0.01.

The analysis of the oil red O staining showed that prolonged photoperiod resulted in increased adipocyte area. Consistently, the mRNA expression of lipogenic marker genes *PPARγ* and *C/EBPα* was significantly increased (p<0.05) in the LP group (Figure 2A). In the LP group, the mRNA expression of lipolysis-related genes *ATGL*, *HSL*, *PPARα*, and *CPT1α*, exhibited a significant decrease (p<0.05) compared to the SP group (Figure 2B). The Western blot results showed that prolonged photoperiod significantly increased PPARγ protein level, while it decreased PPARα and MTR1 protein levels in BSF (p<0.05; Figure 2D). Furthermore, enzyme activities of ATGL and HSL were analyzed by ELISA assay in the two groups of BSF tissues. The findings indicated a significant reduction (p<0.05) in HSL and ATGL enzyme activities within the LP group compared to the SP group (Figure 2E, 2F).

### Influence of prolonged photoperiod on mRNA and protein expression of genes related to lipid metabolism, the activity of lipolytic lipase in liver

**Figure 3:**
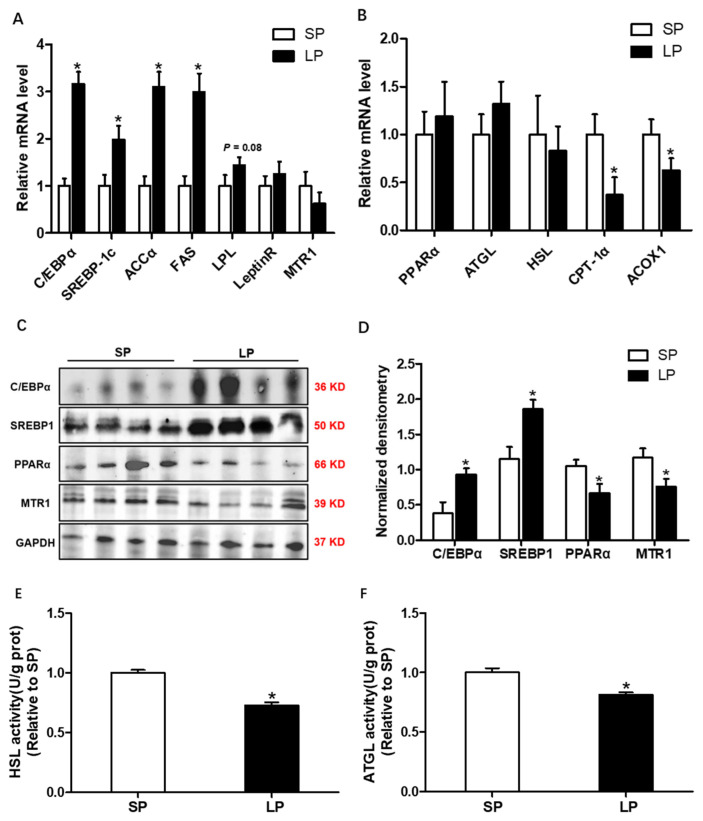
Influence of prolonged photoperiod on mRNA and protein expression of genes related to lipid metabolism, the activity of lipolytic lipase in the liver. (A) The relative mRNA level of lipid synthesis genes *C/EBPα, SREBP-1c, ACCα, FAS, LPL, LeptinR*, and *MTR1* in LP and SP groups. (B) The relative mRNA level of lipolysis genes *PPARα, ATGL, HSL, CPT-1α*, and *ACOX1* in LP and SP groups. (C) Western blot analyses were performed to detect the changes in *C/EBPα, SREBP-1c, PPARα*, and *MTR1*. (D) Densitometric analysis of corresponding proteins in (C) by normalization to GAPDH as an internal control. (E) Hormone-sensitive lipase activity. (F) Adipose triglyceride lipase activity. All values are presented as means± standard error of the mean (n = 12). Representative Western blot is presented. LP, long photoperiod group; SP, short photoperiod group. * p<0.05; ** p<0.01.

The mRNA expressions of lipogenic genes *C/EBPα*, *SREBP-1c*, *ACCα*, and *FAS* were significantly increased (p<0.05; Figure 3A), while the expression level of the lipolysis genes *CPT1α* and *ACOX1* was markedly down-regulated in the LP group (Figure 3B). Western blotting analysis suggested that the protein level of SREBP-1c and C/EBPα were up-regulated, while the protein level of PPARα and MTR1 were decreased in the liver from LP group compared with SP group (p<0.05; Figure 3D). In addition, the activities of HSL and ATGL were significantly lower (p<0.05) in the LP group (Figure 3E, 3F).

### Effects of prolonged photoperiod on the expression of lipid metabolism-related genes, the activity of lipolytic lipase in muscle

**Figure 4:**
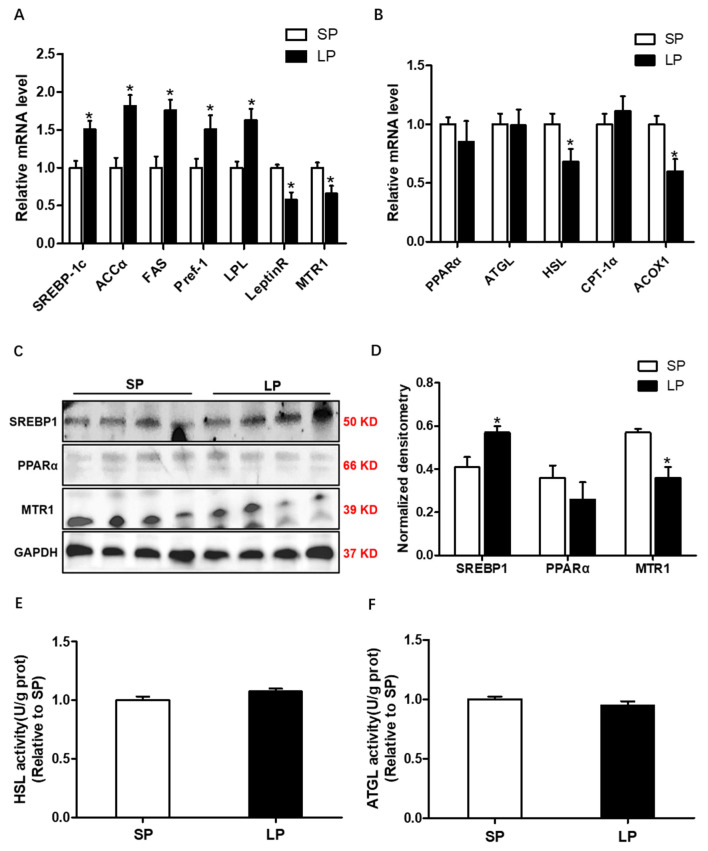
Effects of prolonged photoperiod on the expression of lipid metabolism-related genes, the activity of lipolytic lipase in longissimus dorsi muscle. (A) The relative mRNA level of lipid synthesis genes *SREBP1c, ACCα, FAS, Pref-1, LPL, LeptinR*, and *MTR1* in LP and SP groups. (B) The relative mRNA level of lipolysis genes *PPARα, ATGL, HSL, CPT-1α*, and *ACOX1* in LP and SP groups. (C) Western blot analyses were performed to detect *SREBP1, PPARα*, and *MTR1* changes. (D) Densitometric analysis of corresponding proteins in (C) by normalization to GAPDH as an internal control. (E) Hormone-sensitive lipase activity. (F) Adipose triglyceride lipase activity. All values are presented as means±standard error of the mean (n = 12). Representative Western blot is presented. * p<0.05; ** p<0.01.

The mRNA expression of *LeptinR* and *MTR1* was significantly decreased in the LP group, but the mRNA expression of lipogenic marker genes *SREBP-1c*, *ACCα*, *FAS*, *Pref-1*, and *LPL* was significantly increased in the LP group than that in the SP group (p<0.05; Figure 4A). The mRNA expression of lipolysis-related genes, including *HSL* and *ACOX1* was decreased in the LP group (p<0.05) compared with the SP group (Figure 4B), without a significant change in *PPARα*, *ATGL*, and *CPT1α*. Protein expression levels of PPARα, SREBP-1c, and MTR1 were analyzed by Western blot in BSF (Figure 4C). The results showed that the protein level of SREBP-1c was increased, and decreased MTR1 protein level was observed in the LP group (p<0.05; Figure 4D). Moreover, the activities of HSL and ATGL were no significant differences between the LP group and the SP group (Figure 4E, 4F).

## DISCUSSION

Weaning stress has been shown to reduce feed intake, increase lipolysis, decrease body fat reserves, and impact the growth of piglets [1,3]. Previous studies have demonstrated that weaning stress can be mitigated and feed intake can be increased by changing the component content of the piglet diet [14]. Additionally, apart from nutritional factors, manipulating photoperiod may offer a viable approach to enhance sow productivity and alleviate weaning stress in piglets [15]. It is noteworthy that prior research has reported the potential of LPs to stimulate adipose tissue deposition in animals [6]. However, limited attention has been given to investigate the influence of photoperiod on post-weaning fat metabolism in piglets. Therefore, this study aimed to investigate the effects of prolonged photoperiod on fat metabolism and performance during the entire nursery period for weaned piglets.

Photoperiod is an important environmental factor regulating energy metabolism in animals, and different photoperiod regimes can regulate the performance and fat deposition [16]. It has been demonstrated that the feed intake of weaned piglets in the 23 L:1 D group increased in the second week after weaning compared to the 8 L:16 D group [8]. Niekamp et al [17] fed 4-week-old weaned piglets for six weeks and found that the daily weight gain of piglets in the 16-hour light group was higher than in the 8-hour light group. In this trial, we discovered that the body final weight and ADG of the piglets in the LP group were significantly higher than those in the SP group during 1 to 42 days, but there were no significant differences in ADFI and F/G. This is consistent with previous studies [18], showing that prolonged photoperiod increases body weight without significantly increasing food intake. Coomans et al [19] found that prolonged light disrupted the circadian rhythm and caused weight gain in animals, which may be related to the reduction of energy expenditure. In addition, analysis of ADG and ADFI of weaned piglets at each stage of the trial showed that during the last 2 weeks of the nursery period prolonged photoperiod significantly improved ADG and ADFI of weaned piglets. The fact that prolonged photoperiod has no effect on ADG and ADFI in the early weaning period may be due to the need for piglets to adapt to this circadian rhythm, and also suggested that the effect of prolonged photoperiod duration on growth performance of weaned piglets may occur in the late nursery period.

Multiple hormones have been shown to affect feed intake and weight gain in animals. Some studies have shown that tryptophan can through blood-brain barrier transport, increase serotonin production, and increase feed intake and ADG of weaned piglets [20]. However, Koopmans et al [21] study on weaned piglets showed increased serotonin levels in the experimental group, but no significant differences in ADG and ADFI between the two groups. Meanwhile, it has been found that dietary supplementation of melatonin can significantly improve the ADG and feed conversion rate of piglets weaned in the second week [22]. In addition, it has been reported that feeding GH to growing pigs for 30 to 77 days can increase ADG, but reduce feed intake [23]. The results of this study found that prolonged photoperiod reduced the contents of serotonin, melatonin, and GH in the blood of weaned piglets, while significantly increased ADG, but there was no significant difference in ADFI during the whole test period. The effects of serotonin, melatonin, and GH on the growth performance of weaned piglets are not consistent, and the specific mechanisms of their effects need to be further studied.

In this study, we found that prolonged photoperiod treatment could significantly increase the BF and backfat index of weaned piglets. Previous studies have shown that prolonged photoperiod can increase BF in Jinjiang cattle [6], consistent with our findings. In addition, weaned piglets in the LP group in this study had higher plasma concentrations of TG and NEFA compared to control weaned piglets at 42 d. Consistently, increased TG and NEFA were observed in the liver, BSF, and LDM tissues from weaned piglets compared with the control group. Higher levels of TG and NEFA were linked to decreased enzyme activity of HSL and ATGL in the BSF and liver from the LP group. All these results suggest that prolonged photoperiod affects the growth development and fat deposition of weaned piglets.

The regulation of lipid metabolism has been found to be tightly linked to a number of hormones, the levels of which may be influenced by photoperiod either directly or indirectly. Research has shown that ewes exposed to the longer photoperiod have a tendency to secrete low levels of GH [24]. Compared with an 8-h photoperiod, serum melatonin levels in goats decreased under the LP condition of 16 h [25]. Danilenko et al [26] have shown that seasonal changes in the photoperiod may affect the circadian amplitude and daytime levels of blood serotonin, with plasma serotonin levels higher in summer compared to winter. In the present study, we found that the serum concentrations of melatonin, serotonin and GH were decreased by the LP, which is consistent with previous research findings. In addition, higher levels of melatonin inhibit the accumulation of fatty acids, thereby inhibiting the formation of TG [27,28]. And GH promotes the process of lipolysis [29]. Therefore, prolonged photoperiod decreases the levels of melatonin and GH, increases fat anabolism, and increases the levels of TG and free fatty acids, while significantly decreasing the mobilization of body fat catabolism.

In the present study, we found prolonged photoperiod induced TG accumulation in BSF tissues, as evidenced by the presence of significantly larger adipocyte size in H&E-stained BSF tissues compared to the control group. Consistent with the H&E staining results, the detection of lipid accumulation by prolonged photoperiod was further confirmed by gene expression analysis. In this experiment, prolonged photoperiod treatment significantly up-regulated the expression of *PPARγ* and *C/EBPα* genes and proteins in the BSF tissue, suggesting that lipid uptake and lipogenesis were increased in BSF tissues of weaned piglets. Guerrero-Vargas’ data also showed that the constant light group (24 h continuous light) up-regulated the expression of *PPARγ* mRNA and increased lipid synthesis in rats [30]. *PPARγ* and *C/EBPα* interact to promote lipid synthesis and the expression of genes involved in lipogenesis [31]. In addition, studies have shown that GH may negatively regulate the maturation and accumulation of lipids in adipocytes by decreasing the expression of *C/EBPα* and *PPARγ* [32]. Meanwhile, cells treated with melatonin showed that the expression of *PPARγ*, a specific adipogenesis regulatory gene, was significantly inhibited [33]. This suggests that the reduced levels of GH and melatonin caused by prolonged photoperiod may be partly responsible for the increased expression of the *C/EBPα* and *PPARγ* genes. It is possible that GH and melatonin regulate *PPARγ* expression by multiple pathways, and the specific mechanism remains to be further studied.

The lipogenesis related transcription factor *SREBP-1c* regulates the endogenous production of saturated and monounsaturated fatty acids, including *FAS* and *ACC* [34]. In this study, the mRNA and protein expression of SREBP-1c in the liver and LDM of weaned piglets in the LP group was higher than that in the SP group, suggesting that the increase of TG and NEFA in the liver and LDM tissues of weaned piglets in the LP group may be related to SREBP-1c. In addition, we found that prolonged photoperiod increased the expression of *ACCα* and *FAS* in liver and LDM tissue. This suggests that prolonged photoperiod improves the ability to resynthesise free fatty acids, leading to increased fat accumulation [35]. Ruiz et al [36] found that 18 hours of light exposure increased the expression of *SREBP-1c*, *ACCα*, and *FAS* genes in rat liver compared to 6 and 12 hours of light exposure, which is consistent with the study in this article. In addition, increased melatonin content decreased the expression of *SRBEP-1c* and *FAS* mRNA and reduced lipid accumulation in the mouse liver [37]. Li et al [38] demonstrated that GH inhibited lipid accumulation and lower the expression levels of adipogenic key genes (*SCD1*, *SREBP1*, *PPARγ*, and *C/EBPα*) during adipocyte differentiation. Prolonged photoperiod decreased the secretion of GH and melatonin, increased the expression of adipogenic genes in liver and LDM tissues, and inhibited the process of lipolysis.

Another important factor affecting fat accumulation is lipolysis. Studies have shown that the expression of PPARα and CPT1α proteins and genes of guinea pigs were down-regulated by increasing light exposure (24 h) in liver and adipose tissues [39]. In line with these results, we found that prolonged photoperiod resulted in decreased mRNA expression of the lipolysis gene *CPT1α* in *BSF* and liver tissues. Western blot analysis further showed that prolongation of photoperiod could promote the decrease of PPARα protein expression in liver and BSF tissue. Liu et al [39] found that melatonin treatment markedly upregulated *CPT1A* and *PPARα* gene and protein expression [39]. In addition, we found that prolonged photoperiod led to decreased expression of the lipolysis genes *ATGL* and *HSL* in BSF tissues, and decreased the activity of ATGL and HSL enzymes in liver and BSF tissues, reducing the rate of lipolysis. Studies have demonstrated that melatonin up-regulated the expression of lipolytic genes, such as *HSL* and *ATGL*, and markedly increased lipolysis [40]. In addition, GHs may not directly affect the expression of *ATGL* and *HSL*, but may increase the expression of *ATGL* and *HSL in vivo* through indirect means [35]. The specific mechanism remains to be further studied. Therefore, we suggest that prolonged photoperiod increases fat deposition in weaned piglets during the nursery period by regulating the expression of lipogenic genes and decreasing the expression of lipolysis genes and lipase activity by suppressing the secretion of melatonin and GHs.

## CONCLUSION

The results of this study show that prolonged photoperiod can improve the body weight and ADG of weaned piglets during the nursery period, and increase piglet BF and increase piglet body fat content, and has a tendency to improve ADFI. Our analysis reveals that prolonged photoperiod can affect the blood hormone serotonin, GH, and melatonin level, lipid metabolism related gene expression and lipase activity of weaned piglets, and improve the fat deposition ability of weaned piglets. Collectively, our study revealed that prolonged photoperiod may be an important factor in alleviating weaning stress, and improving performance and body fat reserve in weaned piglets. This study provides a scientific basis for light management of weaned piglets during nursery period.

## Figures and Tables

**Table 1:** Composition and nutrient levels of the weaner diet (as fed)

Items	
Ingredients (%)	
Corn	61.65
Matured soybean meal^1)^	18.00
Flour	5.00
Fermented soybean meal	5.00
Fish meal	2.00
Soybean oil	2.50
White sugar	2.00
Limestone	0.60
CaHPO_4_	1.40
Salt	0.35
Lysine-HCL	5.00
Premix^2)^	1.00
Total	100.00
Nutrients	
Metabolizable energy (MJ/kg)	14.20
Crude protein %	18.20
Digestible lysine %	1.30
Digestible methionine + cystine %	0.72
Total calcium %	0.62
Available phosphorus %	0.59

1) Matured soybean meal: soybean meal through high temperature and high-pressure processing.

2) Ingredients of premix provided per kg of feed: vitamin A, 16,000 IU; vitamin D, 3,000 IU; vitamin E, 50 mg; vitamin K, 3 mg; vitamin B_1_, 5 mg; vitamin B_2_, 12 mg; vitamin B_6_, 13 mg; folic acid, 1.5 mg; nicotinic acid, 60 mg; pantothenic acid, 30 mg; biotin, 0.25 mg; copper, 110 mg (as CuSO_4_·5H_2_O); iron, 200 mg (as FeSO_4_·7H_2_O); manganese, 55 mg (as MnO_2_); zinc, 110 mg (as ZnSO_4_); iodine, 0.7 mg (as KI); selenium, 0.4 mg (as Na_2_SeO_3_·5H_2_O).

**Table 2:** Gene-specific primers for the analysis of pig gene expression

Gene	Accession no.	Primer sequences (5′→3′)	Product size (bp)
*CEBPα*	XM_003127015.4	F: CCAAGAAGTCGGTAGACAAGAACAG	149
		R: GCGGTCATTGTCACTGGTCAG	
*SREBP-1c*	NM_214157.1	F: CCGCTCCTCCATCAATGACAAG	130
		R: CTGGTTGCTCTGCTGAAGGAAG	
*PPARα*	NM_001044526.1	F: AATAACCCGCCTTTCGTCATACAC	97
		R: CCTTGTTCTGGATGCCGTTGG	
*ACCα*	NM_001114269.1	F: AAGAGGTTCCAGGCACAGTCC	142
		R: TCAGCATGTCAGAAGGCAGAGG	
*FAS*	NM_001099930.1	F: CTCCTTCTTCGGGGTCCACTC	103
		R: GTTGATGCCTCCGTCCACAATG	
*LPL*	NM_214286.1	F: ACACAGTTGAGGACACTTGCCATC	116
		R: TCCTGTCACCGTCCAGCCATG	
*ATGL*	NM_001098605.1	F: CTACGAACTCAAGAGCACCATCAC	148
		R: CTTGGAGAGGCGGTAGAGGTTG	
*HSL*	NM_214315.3	F: TTGAAATGCCACTGACTGCTGAC	132
		R: GCTCCTCACTGTCCTGTCCTTC	
*CPT-1α*	NM_001129805.1	F: CGGTTGCTGACGATGGTTATGG	87
		R: GGCAGGAGAACTTGGAAGATATGTG	
*ACOX1*	NM_001101028.1	F: TGAGTCACAGGAAGAGCAAGGAG	148
		R: AAGACAGCGTGGATGGACCTC	
*Leptin R*	NM_001024587.1	F: CTGCTTGTAGACAGTGTGCTTCC	94
		R: TGCTCCAGTCACTCCAGATTCC	
*MTR1*	XM_021078041.1	F: GCTCATCCTCATCTTCACCATCG	91
		R: TGCGTTCCTCAGCTTCTTGTTC	
*Pref-1*	NM_001048187.1	F: GGCATCGTCTTCCTCAACAAGTG	88
		R: GCAGCAGCAGGTTCTTCTTCTTG	
*PPARγ*	NM_214379.1	F: TCTGTGGACCTGTCGGTGATG	90
		R: TGGAGTGGAAATGCTGGAGAAATC	
*SCD1*	NM_213781.1	F: ACTACCATCACAGCACCTTCCTC	102
		R: TTTCATTTCAGGGCGGATGTCTTC	
*ACSL1*	NM_001167629.2	F: AAAGCACATCTTCAAATTGGCACAG	81
		R: ACAGGCTCACTTCGCAGGTAG	
*ADRP*	NM_214200.2	F: GATTGCCATTGCCAACACTTACG	149
		R: CAGTCACAGTAGTCGTCATAGCATC	
*DGAT1*	NM_214051.1	F: GAACCTCATCAAGTACGGCATCC	129
		R: TGGAACGCAGTCACAGCAAAG	
*GAPDH*	NM_001206359.1	F: CAAGTTCCACGGCACAGTCAAG	79
		R: TCGCTCCTGGAAGATGGTGATG	

*C/EBPα*, CCAT enhancer binding protein alpha; *SREBP-1c*, sterol regulatory element binding transcription factor 1; *PPARα*, peroxisome proliferator-activated receptor alpha; *ACCα*, αacetyl-CoA carboxylase α; *FAS*, fatty acid synthetase; *LPL*, lipoprotein lipase; *ATGL*, adipose triglyceride lipase; *HSL*, hormone-sensitive lipase; *CPT-1α*, carnitine palmitoyl transferase 1 alpha; *ACOX1*, acyl-Coa oxidase 1; *Leptin R*, leptin receptor; *MTR1*, melatonin receptor 1; *Pref-1*, proadipocytokine 1; *PPARγ*, peroxisome proliferator-activated receptor gamma; *SCD1*, stearoyl CoA desaturase 1; *ACSL1*, acyl-Coa synthetase long chain family member 1; *ADRP*, adipose differentiation related proteins; *DGAT1*, diacylglycerol o-acyltransferase 1; *GAPDH*, glyceraldehyde-3-phosphatedehydrogenase.

**Table 3:** Effect of photoperiod on growth performance of nursery pigs

Items	Days	SP^1)^	LP^1)^	p-value
BW (kg)	1	8.30±0.13	8.48±0.22	0.510
	14	11.30±0.55	11.00±0.93	0.348
	28	17.71±1.67	17.19±0.27	0.355
	42	24.38±0.51^b^	26.14±0.38^a^	0.011
ADFI (g/d)	1–14	411.61±14.45	390.48±5.33	0.192
	15–28	883.04±21.03	906.25±14.02	0.370
	29–42	1,297.12±14.07^b^	1,354.49±13.49^a^	0.008
	1–42	853.35±15.14	883.60±6.75	0.088
ADG (g/d)	1–14	214.05±9.19	180.18±18.05	0.114
	15–28	457.68±33.50	441.97±20.81	0.694
	29–42	476.73±35.28^b^	639.58±21.81^a^	0.001
	1–42	382.82±12.16^b^	420.58±9.72^a^	0.024
F/G	1–14	1.95±0.09	2.45±0.27	0.104
	15–28	2.04±0.14	2.09±0.08	0.739
	29–42	2.95±0.29^a^	2.14±0.07^b^	0.019
	1–42	2.25±0.07	2.11±0.05	0.131
BF (mm)	1	4.00±0.21	4.25±0.25	0.455
	42	5.16±0.32^b^	6.63±0.32^a^	0.003
BF index^2)^	42	0.21±0.01^b^	0.25±0.01^a^	0.046

Results are expressed as mean±standard error of the mean for SP (n = 12) and LP (n = 12).

BW, body weight; ADFI, average daily feed intake; ADG, average daily weight gain; F/G, a ratio of feed intake to weight gain; BF, Back-fat thickness.

1) LP, long photoperiod group (16 L:8 D); SP, short photoperiod group (10 L:14 D).

2) A ratio of BF day_42_ to BW day_42_.

a,b Means within the same row with different superscripts mean significant difference (p<0.05).

**Table 4:** Blood glucose and lipid fractions of the studied piglets in nursery phase

Items	Days	SP^1)^	LP^1)^	p-value
Glucose (mmol/L)	14	5.25±0.18	6.04±0.44	0.118
	42	4.14±0.60	3.87±0.12	0.679
HDL (mmol/L)	14	1.13±0.04	1.19±0.02	0.212
	42	0.61±0.05	0.72±0.05	0.171
LDL (mmol/L)	14	0.86±0.05	0.79±0.07	0.463
	42	1.62±0.13	1.40±0.17	0.316
CHOL (mmol/L)	14	1.73±0.11	1.52±0.11	0.218
	42	3.16±0.18	2.72±0.27	0.196
TG (mmol/L)	14	0.39±0.02	0.34±0.02	0.108
	42	0.59±0.02	0.65±0.03	0.096
NEFA (μmol/L)	14	0.33±0.02	0.30±0.01	0.164
	42	0.56±0.01	0.57±0.00	0.067

Results are expressed as mean±standard error of the mean for SP (n = 12) and LP (n = 12).

HDL, high-density lipoproteins; LDL, low-density lipoprotein; CHOL, total cholesterol; TG, triglycerides; NEFA, nonesterified fatty acid.

1) SP, short photoperiod group; LP, long photoperiod group.

**Table 5:** Blood hormone levels of the studied piglets in nursery phase

Items	Days	SP^1)^	LP^1)^	p-value
Insulin (mIU/L)	14	115.80±3.39	121.83±3.56	0.240
	42	139.24±4.00	129.74±3.82	0.108
Serotonin (pg/mL)	14	286.42±16.97^a^	207.64±6.68^b^	0.001
	42	356.35±15.4^a^	276.26±5.79^b^	0.001
Melatonin (ng/L)	14	73.40±4.03^a^	58.81±3.45^b^	0.016
	42	113.48±4.1^a^	84.15±1.34^b^	0.000
Leptin (ng/mL)	14	2.10±0.06	2.01±0.03	0.172
	42	2.77±0.07	2.93±0.09	0.196
GH (μg/L)	14	24.17±0.30^a^	21.30±0.11^b^	0.000
	42	30.64±0.69^a^	26.62±0.63^b^	0.001

Results are expressed as mean±standard error of the mean for SP (n = 12) and LP (n = 12).

GH, growth hormone.

1) SP, short photoperiod group; LP, long photoperiod group.

a,b Means within the same row with different superscripts mean significant difference (p<0.05).
